# Classifying the lifestyle status for Alzheimer’s disease from clinical notes using deep learning with weak supervision

**DOI:** 10.1186/s12911-022-01819-4

**Published:** 2022-07-07

**Authors:** Zitao Shen, Dalton Schutte, Yoonkwon Yi, Anusha Bompelli, Fang Yu, Yanshan Wang, Rui Zhang

**Affiliations:** 1 College of Science and Engineering, University of Minnesota, Minneapolis, USA; 2 Department of Pharmaceutical Care and Health Systems, University of Minnesota, Minneapolis, USA; 3 Edson College of Nursing and Health Innovation, Arizona State University, Phoenix, USA; 4 Department of AI and Informatics, Mayo Clinic, Rochester, USA; 5 Institute for Health Informatics, University of Minnesota, Minneapolis, USA

**Keywords:** Natural language processing, Machine learning, Electronic health records, Deep learning, Alzheimer’s disease, Clinical text classification

## Abstract

**Background:**

Since no effective therapies exist for Alzheimer’s disease (AD), prevention has become more critical through lifestyle status changes and interventions. Analyzing electronic health records (EHRs) of patients with AD can help us better understand lifestyle’s effect on AD. However, lifestyle information is typically stored in clinical narratives. Thus, the objective of the study was to compare different natural language processing (NLP) models on classifying the lifestyle statuses (e.g., physical activity and excessive diet) from clinical texts in English.

**Methods:**

Based on the collected concept unique identifiers (CUIs) associated with the lifestyle status, we extracted all related EHRs for patients with AD from the Clinical Data Repository (CDR) of the University of Minnesota (UMN). We automatically generated labels for the training data by using a rule-based NLP algorithm. We conducted weak supervision for pre-trained Bidirectional Encoder Representations from Transformers (BERT) models and three traditional machine learning models as baseline models on the weakly labeled training corpus. These models include the BERT base model, PubMedBERT (abstracts + full text), PubMedBERT (only abstracts), Unified Medical Language System (UMLS) BERT, Bio BERT, Bio-clinical BERT, logistic regression, support vector machine, and random forest. The rule-based model used for weak supervision was tested on the GSC for comparison. We performed two case studies: physical activity and excessive diet, in order to validate the effectiveness of BERT models in classifying lifestyle status for all models were evaluated and compared on the developed Gold Standard Corpus (GSC) on the two case studies.

**Results:**

The UMLS BERT model achieved the best performance for classifying status of physical activity, with its precision, recall, and F-1 scores of 0.93, 0.93, and 0.92, respectively. Regarding classifying excessive diet, the Bio-clinical BERT model showed the best performance with precision, recall, and F-1 scores of 0.93, 0.93, and 0.93, respectively.

**Conclusion:**

The proposed approach leveraging weak supervision could significantly increase the sample size, which is required for training the deep learning models. By comparing with the traditional machine learning models, the study also demonstrates the high performance of BERT models for classifying lifestyle status for Alzheimer’s disease in clinical notes.

## Background

Alzheimer’s disease (AD) is the most common cause of dementia, accounting for 60 to 80 percent of all dementia cases [1]. Around 5.8 million Americans were living with AD in 2020, and this number is expected to increase to approximately 14 million by 2050 [2]. Currently, no treatments can cure AD, but different lifestyle statuses have been associated with a substantially reduced risk for AD with inconsistent findings. For example, high levels of physical and cognitive activity showed the strongest associations with reduced AD risk ranging from 11 to 44% [3]. Mul tiple lifestyle modifications, including physical activity, no-smoking, light-to-moderate alcohol consumption, cognitive activities, and high-quality diets were correlated with a 60% decreased risk for AD [4]. Fur thermore, the Finnish Geriatric Intervention Study to Prevent Cognitive Impairment and Disability (FINGER) found that people at high risk of developing AD showed improvements in their cognitive abilities following 2 years of lifestyle changes [5]. These findings led to the launch of multi-lifestyle intervention trials globally, such as the U.S. Pointer [6]. Few Randomized Controlled Trials (RCTs) have the resources of the U.S. Pointer to be able to enroll a large sample. Hence, alternative, innovative, scalable, and cost-effective approaches to the use of electronic health records (EHRs) to establish causal effects are critically needed.

Since 2009 when the Health Information Technology for Economic and Clinical Health Act (HITECH Act) was enacted [7], EHRs have been adopted exponentially. Consequently, studies using EHRs have increased dramatically and have been acknowledged as a way of enhancing patient care and promoting clinical research [8–11]. EHR document information obtained during healthcare delivery, including detailed explanations of the occurrence, treatment, and progression of diseases. Secondary analysis of observational EHR data has been widely used in multiple clinical domains [12].

A study showed that a large portion of lifestyle information was documented in EHRs in the unstructured narrative or mixed format rather than in the structured data [13]. The nature of EHRs making the lifestyle status-related information be difficult to process and obtain desired information. To overcome this difficulty, natural language processing (NLP) techniques have been used to show promising results in extracting pertinent information from unstructured data for clinical research. For example, in our previous study, we demonstrated that extracting lifestyle factors using standard terminologies such as Unified Medical Language System (UMLS) and the existing NLP model (MetaMap) was feasible and reliable [14]. Our previous studies used standardized rule-based NLP models without the aid of annotated data. Besides, we demonstrated the feasibility of using NLP methods to automatically extract lifestyle factors from EHRs in our latest research [15]. Previously [15], conventional machine learning methods such as the random forest, support vector machine (SVM), conditional random field, logistic regression, bagged decision trees, and K-nearest Neighbors have been used for extracting lifestyle factors related to excessive diet, physical activ ity, sleep deprivation, and substance abuse. However, some limitations included working with a small-sized annotated corpus mainly due to the labor-intensive process of developing the corpus [15].

Recently, more advanced neural network-based representations, such as Bidirectional Encoder Representation from Transformers (BERT) [16], have further improved performance in multiple NLP tasks, such as question answering. BERT models have been further applied in the biomedical domain, and their variants have been pre-trained on various biomedical corpora, such as biomedical literature and clinical records (e.g., MIMIC [17]), to gain a deep representation of biomedical information. In general, these domain-specific BERT models have shown promising performance in clinical applications [18–20]. For instance, Lee et al. [21] presented that Bio BERT, pre-trained on large-scale biomedical corpora, significantly exceeds the standard BERT model on some popular biomedical NLP tasks, i.e., named entity recognition, relation extraction, and question answering. Michalopoulos et al. [22] also showed similar results by comparing the general BERT model with their proposed domain-specific model, UMLS BERT.

On the other hand, BERT based model requires a large amount of labeled training data for achieving better performance. To reduce human efforts to generate annotations, weak supervision is one approach that trains machine learning models using weak labels generated by rule-based methods. Previously, we [23] have demonstrated the feasibility of using weak supervision and deep representation using word embeddings for clinical text classification tasks. This approach involved training word2vec models on clinical notes from the Mayo Clinic and used these representations to train Convolutional Neural Networks and MLP Neural Networks. We describe an approach using pre-trained contextual embeddings and demonstrate improvements over the baseline models.

There have been several clinical applications utilizing attention-based models, such as BERT and Bio BERT, with weak or distant supervision to extract information from unstructured clinical notes. Specifically, in terms of the NER task, Fries et al. [24] presented Trove, a novel framework combining weakly supervised entity classification using medical ontologies and expert-generated rules with a Bio BERT model. This framework demonstrated promising performance for classifying risk factors of COVID-19. Similarly, Chen et al. [25] proposed BOND, a NER task pipeline with a two-stage training process: a BERT training on distant labels with an early stop followed by a self-training strategy on fine-tuning the model. Besides NER tasks, the fashion of combining the weak supervision with BERT models was also proved to be very effective on other clinical NLP tasks, such as medication regimen extraction [26]. By comparison, our approach uses only the raw text with weak labels generated by simple rules as input to various models, meaning our pipeline is much more simple and direct than those proposed in the aforementioned studies. We also consider a wider collection of biomedical BERT variants to assess how each performs on clinical data.

Hence, the objective of this study was to compare the performance of the rule-based model, conventional machine learning models, and BERT models with weak supervision on classifying the status of lifestyle from the clinical notes with the unstructured format. Our contributions include: (1) evaluating traditional machine learning models and state-of-the-art biomedical and clinical BERT models on the classification of the lifestyle status-related sentences in clinical notes of patients with AD, (2) using weak supervision to overcome the burdensome task of creating a hand-labeled dataset, and (3) comparing models’ performance when training on various proportions of the weakly supervised data.

## Methodology

**Fig. 1:**
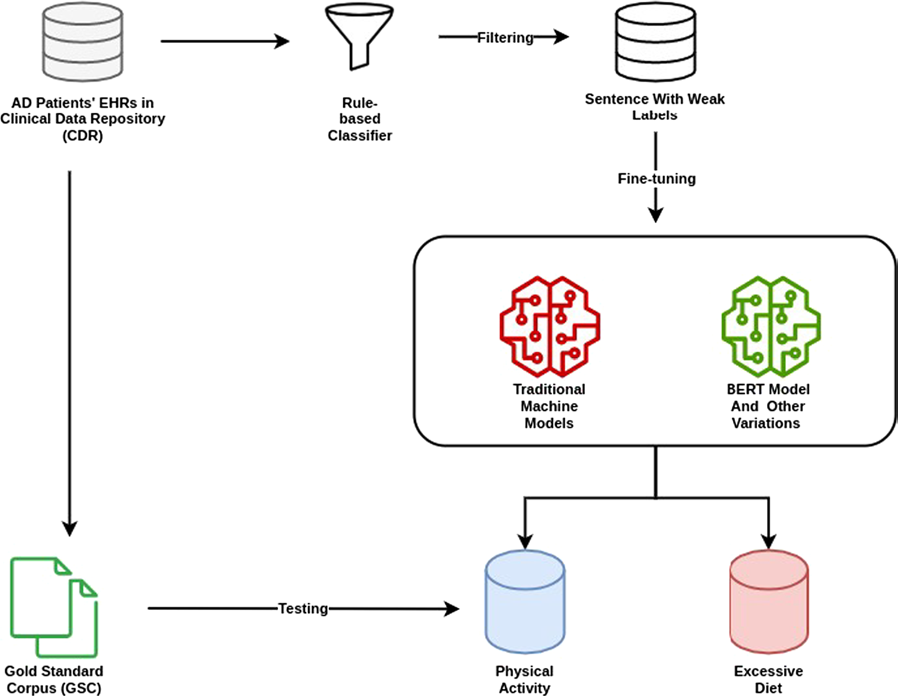
Overview of the study design

We conducted our experiments on two categories of lifestyle status: physical activity and excessive diet. For each case study, we followed the same steps: (1) collecting clinical notes from patients with AD; (2) applying the rule-based NLP classifier to assign weak labels to the lifestyle-based sentences; (3) Training three traditional machine learning models and fine-tuning BERT models on the data with weak labels for each case study described below; (4) manually annotating a small portion of the selected sentences to develop the Gold Standard Corpus (GSC); 5) evaluating the performances of various machine learning models and BERT models in the GSC. Figure1 demonstrates the whole workflow for this study.

### Data source

The clinical notes were sourced from the Clinical Data Repository (CDR) of the University of Minnesota (UMN). More than 180 million clinical notes are currently held by the aforementioned CDR, containing more than 2.9 million patients from 8 hospitals and more than 40 local clinics. All of the records were in English. Approval was obtained to access the EHRs for patients with AD from the Institutional Review Board (IRB). Then, by using the online UMLS Metathesarus browser based on our previous work, we manually collected all concept unique identifiers (CUIs) associated with physical activity and excessive diet [15]. After collecting all AD clinical notes labeled with related CUIs in a database, each note was broken down to the sentence level. The cleaning included removing repeated sentences, stripping of punctuation and extra spaces, and setting the whole sentence to lowercase.

To generate the GSC, three annotators independently annotated 50 sentences for each case study using INCEpTION [27], a semantic annotation platform. In total, 200 sentences were annotated for physical activity and 88 sentences were annotated for excessive diet. Based on the presence of the lifestyle status entities of interest on the GSC, labels were assigned at sentence level for all selected sentences. Physical activity, we set labels as “yes” or “no” (“no” indicates physical inactivity) to sentences. The category of “physical inactivity” does not include sentences with “no physical activity mentions.” For excessive diet, sentences were assigned as “high calorie diet,” “high salt diet,” “high fat diet,” “normal diet,” and “non-specific abnormal” (indicating no clear diet mentions). Since most sentences had explicit mentions of the lifestyle status, they could be distinguished quickly based on semantic understandings. Hence, for any pair of the three annotators, Cohen’s Kappa score reached 1. The entity names and their example can be found in Table 1. Note that the GSC and training data with weak labels were mutually excluded by ensuring their note ids were not overlapping.

**Table 1:** Example sentences with weak labels for excessive diet and physical activity

Category	Class	Sentence example
Excessive diet	High fat diet	Pt is having fatty food
	High calorie diet	He had token high calorie diet for 2 weeks
	High salt diet	His current diet contains too much food with high salt
	Normal diet	She backs to normal diet
	Non-specific abnormal	She has no knowledge of salt restrictions
Physical activity	Physical activity	Pt has increase regular physical activity
	Physical inactivity	He didn’t maintain daily exercise

### Weak supervision

For generating weak labels for the sentences, we utilized a rule-based classifier written in Python. This rule-based classifier was based on keyword mentions by considering tokens within a neighborhood of the keywords. Keywords for excessive diets were ‘normal,’ ‘high calorie diet,’ ‘high fat diet,’ and ‘high salt diet.’ For physical activity, the keywords included ‘active,’ ‘activity,’ ‘inactive,’ and ‘inactivity.’ A window of 5 terms immediately before the search term was used.

For both excessive diet and physical activity, a “1” would be returned if a match was found and a “0” if not. If a negation term was found within the window, a “− 1” would be returned for determining the final classification. More specifically, for physical (in)activity, in cases where a “− 1” was returned, the final class assignment was for the opposite class (e.g. opposite class for “physical activity” − 1 is “physical inactivity”). For diet, negation of “normal diet” counted towards “non-specific abnormal”, because the other classes were abnormal, negation did not necessarily mean normal and so, for simplicity, the negation of any of the “high X diet” rows were assigned “0”. There was an additional step used to check if the patient was on a normal diet or not. If any of the terms ‘can’, ‘resume’, ‘begin’, ‘start’, ‘going’, ‘may’ were in the window before “normal diet”, a “4” was returned for “nonspecific abnormal.” Furthermore, the only instances where negation of the term occurred was in the class ‘normal diet’, in which case the classification was ‘nonspecific abnormal. Each sentence was assigned to one class only. The same model later would be used on the GSC for comparison.

### Model

With transfer learning, a model can be trained on a large open-domain data set, then fine-tuned on a smaller domain-related corpus based on the task’s requirements. Some benefits of transfer learning include how, for relatively high performance, relatively less annotated data is required during the process of fine-tuning after pre-training on large amounts of unlabelled corpora. In this study, we evaluated six BERT models, including: BERT base model [28], PubMedBERT (pre-trained on the abstracts and full text of biomedical literature) [20], PubMedBERT (pre-trained on only abstracts) [20], Bio BERT [21], Unified Medical Language System (UMLS) BERT [22], and BioclinicalBERT [19]. Fine-tuning BERT models constitute a form of transfer learning. Besides the BERT base model, the other BERT variations can be classified into two groups, which are biomedical focus and clinical focus, based on their domains. One group was biomedical-specific. PubMedBERT (Abs+Ft) was pretrained from scratch using the abstracts and full text of PubMed articles, which included approximately 16.8 billion words (107 GB). The PubMedBert (Abs) model pre-trained from scratch using the abstracts alone, which included 3.2 billion words (21 GB). Similarly, BioBERT was initialized from Base BERT then additional pre-training was done using PubMed abstracts and PubMed Central full-text articles. The remaining two variations of BERT models were clinical domain-specific BERT models. Their training dataset includes Medical Information Mart for Intensive Care III (MIMIC III) dataset [17], besides open-domain training dataset. The MIMIC III corpus has approximately 0.5 billion words (3.7 GB). Note that the Bio-clinical BERT model was pre-trained starting from BioBERT, and the UMLS BERT model was initialized from bioclinical BERT and further pre-trained using additional information from CUIs and UMLS semantic types during the Masked Language Modeling portion of pretraining.

For comparing BERT models’ performance, traditional machine learning models were used as baselines. Specifically, support vector machine (SVM), logistic regression, and random forest. Those conventional machine learning have been successfully applied in several clinical NLP tasks. The feature set used the bag-of-words representation method for generating features for those three machine learning models, including unigrams, bigrams, and trigrams. In addition, term frequency-inverse document frequency (TF-IDF) was applied for adding weights on that *n*-gram-related features. The rule-based model used in weak supervision would also be used for comparison.

### Training and evaluation

For both case studies, we split the sentences into training (90%), validation (10%) in the weak-labeled corpus. Random seeds for train and test set splitting was kept the same for all models during all three runs. Seeds were chosen as 24, 48, and 128. The final result was calculated for each model by taking the average of three results for each metric. First, we trained the machine learning models and fine-tuned BERT models on the training dataset and evaluated on the validation set. The parameters for all traditional machine learning models were found using a fivefold CV with a grid search over the parameter space, including but not limited to kernel-related parameters for SVM (i.e., gamma), penalty related parameters for logistic regression (i.e., penalty type), and tree-related parameters for random forest (i.e., number of trees). The final parameters used for SVM were C = 10 and kernel = ‘linear’. For logistic regression: C = 100, and penalty = ‘12’. For random forest: n estimators = 10, criterion = ‘gini’, and min sample split = 2.

For all BERT models, the team fine-tuned them on training data for ten epochs with a learning rate of 2 * 10^*−*5^. While keeping the trade-off with efficiency in mind, we picked a batch size of 512 for the case study on physical activity. The batch was set as 64 for the excessive diet case study. We used a dropout layer for regularization purposes and a fully connected layer in the end. The dropout rate was set to be 0.3 and Adam was used for optimization. The cross-entropy loss function was used as the loss function. In terms of padding, we padded the sentence to length 50 since the majority of sentences were shorter. The best models in the validation set were further evaluated on the GSC.

## Results

### Corpus statistics

We here describe the corpora for the two case studies to evaluate BERT models’ effectiveness with weak supervision.

#### Case 1: physical activity

We collected 23,559 sentences by searching for physical activity related CUIs from the database. After removing duplicates, there were 12,086 sentences left. A large proportion of the duplicate sentences were related to “physical inactivity” and were largely from common phrases that would be populated into patient notes. Of all the sentences, 11,571 (95.7%) sentences were assigned as “physical activity,” and 515 (4.3%) mentions were “physical inactivity.” In the GSC, the number of sentences in the category of physical activity and physical inactivity is 78 and 122, respectively.

#### Case 2: excessive diet

In total, 886 sentences were used as training data with weak labels. Training data were distributed as 300 (33.8%), 153 (17.4%), 133 (15%), 250 (28.2%), and 50 (5.6%) respectively, for “high calorie diet,” “high fat diet,” “high salt diet,” “normal diet,” and “nonspecific abnormal.” In the GSC, the corresponding numbers were 18, 20, 20, 18, and 12, respectively.

### Computing power and time

During the experiment, all machine learning models were conducted on a cluster of 16 Intel® Xeon® Platinum 8268 Processors with 1 core and all BERT models were implemented on one NVIDIA® GRID V100D-32D GPU. In terms of the computing time, for excessive diet, the process took 2.87 s, 20.69 s, and 3.61 s for logistic regression, random forest, and SVM. Due to the complexity of BERT models, the average fine-tuning time was about 37.39 s. For physical activity, since the training data size was ten times larger than the first case study, all training time increased. For logistic regression, random forest, and SVM, their training time was 8.51 s, 117.84 s, and 320.22 s, respectively. For BERT models, the average fine-tuning time was about 429.08 s, over ten times longer than the first case study.

### Model performance

As shown in Table 2, in terms of the weighted average for physical activity, the UMLS BERT model performed the best with precision, recall, and macro F-1 score 0.93, 0.93, and 0.92, respectively. The best machine learning models were logistic regression and SVM, with the same precision, recall, and macro F-1 scores of 0.91, 0.89, and 0.89. The UMLS BERT model metric was 2%, 5%, and 3% higher than the baseline SVM and logistic regression models. Besides Bio-clinical BERT, the other BERT models’ performance did not exceed the baseline models. Regarding the class “physical activity”, the machine learning models, SVM and logistic regression had perfect precision but had relatively poorly recall. The UMLS BERT had better performance with recall and F1 0.98 and 0.94. All of the BERT models outperformed the machine learning models in recall and F1 score. However, for the “physical inactivity” class, BERT models performed better in precision instead of recall. UMLS BERT still delivered the highest precision and F1 score, which was 0.96 and 0.89, respectively. No BERT models outperformed traditional machine learning models in recall for this class. All models exceed the rule-based method in F1 for all scenarios.

**Table 2:** Comparison of results for models for the physical activity case study

Model	Weighted avg			Physical active			Physical inactivity		
	Precision	Recall	F1	Precision	Recall	F1	Precision	Recall	F1
Rule-based	0.88	0.53	0.62	0.87	0.32	0.47	0.89	0.85	0.87
Logistic regression	0.91	0.89	0.89	**1.00**	0.81	0.90	0.77	**1.00**	0.87
Random forest	0.89	0.88	0.88	0.95	0.85	0.89	0.80	0.93	0.86
SVM	0.91	0.89	0.89	**1.00**	0.82	0.90	0.79	**1.00**	0.88
BERT base	0.90	0.90	0.89	0.89	0.94	0.92	0.91	0.82	0.86
Bio BERT	0.89	0.89	0.89	0.91	0.92	0.91	0.88	0.85	0.60
PubMed BERT (Abs)	0.90	0.89	0.89	0.89	0.95	0.92	0.92	0.80	0.85
PubMed BERT (Abs + Ft)	0.88	0.88	0.87	0.86	0.95	0.90	0.91	0.76	0.82
Bio-clinical BERT	0.91	0.91	0.91	0.91	0.95	0.93	0.91	0.86	**0.89**
UMLS BERT	**0.93**	**0.93**	**0.92**	0.91	**0.98**	**0.94**	**0.96**	0.84	**0.89**

*Bold numbers indicate best performance in each column

In the use case on the excessive diet, the machine learning model with the best weighted average was the random forest, with precision, recall, and F1 score 0.89, 0.86, and 0.86. Bio-clinical BERT outperformed the random forest in all metrics. Hence, Bio-clinical BERT was the model with the best performance overall. For the class “normal diet”, Bio-clinical BERT outperformed the other models in precision. Bio BERT had the highest F1 score, which was 0.74. All BERT models had higher precision than three machine learning models had. For the class “nonspecific abnormal”, the Bio-clinical BERT model had the highest recall and F1 scores. Compared with the machine learning models, BERT models had better scores in recall and F1. In addition, all models had nearly perfect precision, recall, and F1 scores for “high calorie diet” and “high salt diet”. For the class “high fat diet”, all BERT models had better performance than the three machine learning models. The rule-based model had good performance in most of the metrics for this case study, except for the precision of the class “normal diet” and the recall of the class “nonspecific abnormal.” It had better performance than most machine learning models and some of the BERT models in some scenarios. The full results can be found in Table 3.

**Table 3:** Comparison of results for models for the excessive diet case study

Model	Weighted avg			Normal diet			High calorie diet		
	Precision	Recall	F1	Precision	Recall	F1	Precision	Recall	F1
Rule-based	0.91	0.86	0.87	0.52	**0.92**	0.67	0.94	0.94	0.94
Logistic regression	0.88	0.85	0.85	0.50	0.83	0.63	**1.00**	0.94	0.97
Random forest	0.89	0.86	0.86	0.52	0.89	0.66	**1.00**	0.94	0.97
SVM	0.88	0.85	0.85	0.50	0.83	0.63	**1.00**	0.94	0.97
BERT base	0.91	0.91	0.91	0.66	0.75	0.70	**1.00**	0.98	0.99
Bio BERT	0.92	0.92	0.92	0.71	0.78	**0.74**	**1.00**	0.98	0.99
PubMed BERT (Abs)	0.91	0.90	0.90	0.59	0.81	0.68	**1.00**	**1.00**	**1.00**
PubMed BERT (Abs + Ft)	0.90	0.90	0.90	0.63	0.64	0.62	**1.00**	**1.00**	**1.00**
Bio-clinical BERT	**0.93**	**0.93**	**0.93**	**0.73**	0.75	0.73	**1.00**	**1.00**	**1.00**
UMLS BERT	0.92	0.92	0.92	0.72	0.69	0.70	**1.00**	**1.00**	**1.00**

	High fat diet			High salt diet			Nonspecific abnormal		
	Precision	Recall	F1	Precision	Recall	F1	Precision	Recall	F1
Rule-based	**1.00**	0.90	0.95	**1.00**	0.95	0.97	**0.92**	0.61	0.73
Logistic regression	0.95	0.95	0.95	**1.00**	**1.00**	**1.00**	0.82	0.50	0.62
Random forest	0.95	0.95	0.95	0.98	**1.00**	0.99	0.87	0.50	0.64
SVM	0.95	0.95	0.95	**1.00**	**1.00**	**1.00**	0.81	0.50	0.62
BERT base	0.98	**1.00**	0.99	**1.00**	**1.00**	**1.00**	0.82	0.74	0.78
Bio BERT	0.98	0.98	0.99	**1.00**	**1.00**	**1.00**	0.85	0.80	0.82
PubMed BERT (Abs)	**1.00**	0.98	0.99	**1.00**	**1.00**	**1.00**	0.84	0.65	0.73
PubMed BERT (Abs + Ft)	**1.00**	0.98	0.99	**1.00**	**1.00**	**1.00**	0.77	0.76	0.76
Bio-clinical BERT	**1.00**	0.98	0.99	**1.00**	**1.00**	**1.00**	0.85	**0.83**	**0.84**
UMLS BERT	**1.00**	**1.00**	**1.00**	**1.00**	**1.00**	**1.00**	0.81	0.81	0.81

*Bold numbers indicate best performance in each column

**Fig. 2:**
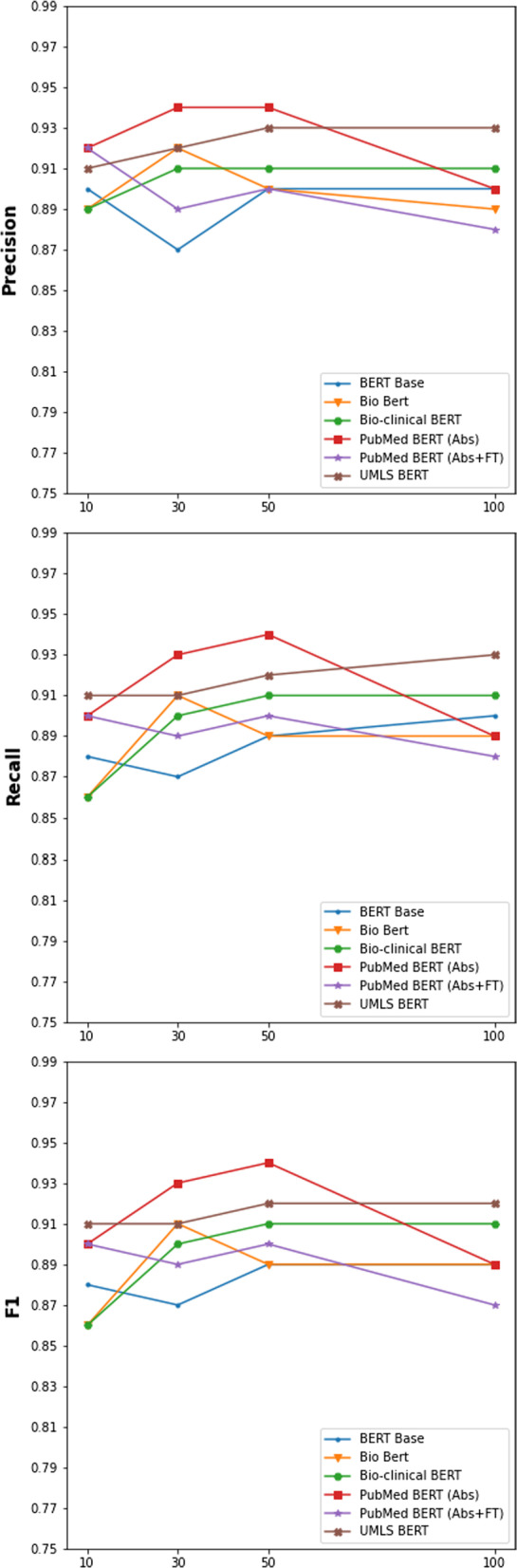
Results for training models on portions of physical activity data

Figure 2 demonstrated all models’ performance changes when training/fine-tuning with different proportions of the weakly supervised data changed. When ratios of the weakly supervised data increased from 10 to 30%, half of the models improved their performance in precision and F1. In terms of recall, only two models’ performance dropped. From the range 30% to 50%, only Bio BERT had a significant drop on all three model metrics. The rest models had better performance or stayed the same for all metrics. With the number of the weakly supervised data doubled from 50 to 100%, regarding F1 and recall, except for PubMed BERT (Abs), PubMed BERT (Abs + Ft), the other models’ performance kept increasing or stayed the same. Meanwhile, no models had a better score for precision, and a few of the models’ performances even dropped.

## Discussion

The wide adoption of EHRs in the healthcare system generates big data about the healthcare delivery of patients. The rapid advancement of artificial intelligence (AI) methods and computational resources provide an alternative way to cost-effectively consider the impact of the lifestyle status on AD using rich EHR data [29]. In this study, we demonstrated the feasibility of using BERT models with weak supervision to classify the lifestyle status for AD in clinical notes. The approach described in this study can be further extended to another status of lifestyle factors, accelerating our investigation on the roles of the lifestyle status on AD.

To effectively train deep neural network models, extensive training data is required. Similar to other studies [23], weak supervision can generate sufficiently large training data by leveraging a rule-based NLP system without requiring additional human efforts. After observing the performance of the rule-based classifier, which was used for weak supervision, we could see weakly labeled training data contain a certain noisy label. However, our finding demonstrated BERT models’ promising performance on weakly labeled data on two classification tasks for the lifestyle status. By comparing the performance of BERT models on different proportions of the weakly supervised data, BERT models show some robustness to noise in the data. Figure 2 shows that after the proportions of the weakly supervised data went beyond 30%, the trend on three model metrics was not decreasing for most BERT models.

For each BERT model, their performances on different case studies were still distinguishable, but BERT models with clinical focus had better performance overall. For example, in the physical activity, UMLS BERT outperformed the rest models; The latter model performed the best in classifying the excessive diet. In addition, all BERT models were robust to a dataset with imbalanced classes. Also, most BERT models outperformed traditional machine learning models in the weighted averages of the three metrics. It demonstrates the advantage of using BERT models on classification tasks for the status of lifestyle factors. On the other hand, unsurprisingly, BERT models’ computing time was usually ten times longer than the traditional machine learning models, even under GPU usage.

For physical activity classification, we noticed a general trend for the BERT models’ F1-scores to improve as a larger portion of the weakly labeled data was used for training. Typically, there wasn’t much improvement in F1-scores when 100% of the data was used compared to when only 50% of the data was used. We did notice that both of the PubMed BERT models actually had decreasing F1-scores when the proportion of the original data set used for training increased from 50 to 100%. All of the other BERT models either showed slight improvement or no improvement. We suspect this might be due to the pre-training procedures for these models. The PubMed BERT models were pre-trained exclusively on PubMed abstracts and/or text, which is a particularly academic style of language that would not be expected to be seen often in patient notes. All of the other models had additional pre-training from Base BERT using biomedical text and so started from a language model that would be closer to “typical” language that might be found in patient notes. When selecting BERT models, consideration should be given to the pre-training and, if applicable, continued pre-training corpora and what would most closely align with the dataset in question. Our results also suggest that experimenting with different proportions of weakly labeled data can be a useful exercise to find an optimally performing model. The BERT variant and proportion of weakly labeled data should be treated as hyperparameters for individuals to experiment with to determine the best values for their use-case.

Due to the small number of training examples of excessive diet, we did not believe that a similar set of experiments using portions of the weakly labeled data would produce meaningful results. Even using 50% of the data would result in only 440 training examples.

During error analysis, we compared instances where the BERT model incorrectly classified a test case, but the traditional model correctly classified a test case, and vice versa. For the exercise test cases where the best traditional model, SVM, was incorrect but the best BERT model, UMLS BERT, was correct were all “physical inactivity” cases. The SVM model struggled to correctly determine the label if either (1) the sentence was long with multiple clauses or (2) the phrase “physically inactive” was present; in these cases, the UMLS BERT model made the correct classification. Interestingly, the cases that the UMLS BERT model missed but the SVM model correctly labeled were mostly “activity” sentences. The prevailing issue for the BERT model was sentences where some form of “activity” was present, but a negating term occurred elsewhere in the sentence in relation to another term. For example, UMLS BERT missed but SVM correctly labeled the sentence, “PT continues to be physically active without doing any aerobic exercise outside of cardiac rehab.”

We also examined test cases that the rules-based classifier correctly labeled but UMLS BERT or the SVM model labeled incorrectly, and vice versa. The majority of cases that the BERT and SVM models were able to capture but the rules-based classifier was not, were due to terms appearing outside of the five token window that we set for the rules-based classifier. Because BERT is able to ingest the entire sentence it does not have the issue of terms falling outside the window and it can consider the entire context. Similarly, the SVM model is not bound by a window. Cases that the rules-based classifier succeeded where the SVM model failed were, as above, due to the phrase “physical inactive” since there were rules explicitly written to handle this exact string. The cases where UMLS BERT failed but the rules-based classifier succeeded also primarily contained negations of “activity”. As mentioned above, the BERT model struggled with these cases however, the rules-based classifier had rules that were explicitly written to handle negation and so could more accurately determine the proper class in cases where activity was negated with another term.

For the diet test cases where the best traditional model, random forest, was incorrect but the best BERT model, Bio-clinical BERT, was correct were mostly “nonspecific abnormal” cases. The most noticeable pattern was the random forest model couldn’t reliably detect phrases that suggested the patient was not currently consuming a normal diet (e.g., “can start”, “can resume,” etc.). The cases where Bioclinical BERT was incorrect but the random forest was correct were all “Nonspecific Abnormal” cases. Similar to the case for the exercise cases, Bio-clinical BERT tended to pick up on negation terms in other parts of the sentence. For example, the sentence “she needn’t worry about her calcium if she is eating and drinking a normal diet” was incorrectly labeled as “nonspecific abnormal” by Bio-clinical BERT but correctly labeled as “normal diet” by the random forest model.

Similarly for excessive diet, we compared the rules-based classifier against Bio-clinical BERT and the random forest model. Similar to the BERT model above, the rules-based model tended to catch cases where phrases that suggested the patient was not currently consuming a normal diet but could begin to do so, (e.g. “can start”, etc.) while the random forest model often failed to correctly classify these cases. Of the cases correctly labeled by the rules-based classifier but incorrectly labeled by BERT, both cases were “nonspecific abnormal”. We suspect this is due to the careful consideration given to crafting the rules for this particular class and, as we saw above, the BERT model struggled to reliably label. Thus, compared to the rules-based classifier, the BERT model often missed instances non-specific diets that the patient was or was not consuming while the rules-based classifier tended to classify them properly.

## Limitations

Our study has some limitations, while our performance metric was relatively high (0.92–0.93) for both case studies. For example, the size of the GSC is relatively small, especially for excessive diet. Furthermore, the highly imbalanced class in the case study on physical activity could be a limitation. The imbalanced ratio was about 1:19 between the number of sentences of “Physical Activity” and “Physical Inactivity.” For the excessive diet, the dataset size, which was about 886 sentences, was still relatively small. In addition, the training samples were distributed unevenly among all the diet classes. The issue of the lack of data diversity was another major drawback. For the physical activity case study, we saw rule-based classifier used for weak supervision has the worst performance among all models, which means using keywords could not be enough. However, for models considering contextual information, increasing the training data size could not significantly improve the model’s performance. We believe that this is because the variation between sentences was relatively small. Therefore, models could capture the most patterns without using all data. For excessive diet, this issue became more serious. Almost all models had nearly perfect performance in some classes, including the rule-based classifier. This result indicated the data was not diversified enough, and the pattern was easy to capture. For example, many mentions on a diet were in the format “high xxx diet/food”. The lack of diversified data might be due to the single data resource, which might contain some generic templates on some level.

## Future work

Collectively, our findings have significant implications for further lifestyle research in AD. They provide a methodology to use unstructured EHR data to enhance the strengths of association between the lifestyle status and AD risk, and allow the simultaneous examinations of multiple lifestyle status and their interactive/synergistic effects on cognitive changes and AD risk. Besides, they provide an approach for future casual modeling of lifestyle changes on clinical outcomes in AD. Since EHRs offer a potential source of data, they can be evaluated and defined to address questions that aim to measure the causal effect of intervention or exposure on the outcome of interest. Unlike RCTs that are time-consuming and expensive to carry out and have minimal generalizability, conducting studies using EHR data is scalable and affordable. Developing causal modeling methods using EHR data will allow large-scale and pragmatic trials. In addition, currently, we did not assess how the lifestyle information is documented in the structure data. In the future, we will compare the representation of lifestyle information in the structure data versus the unstructured notes.

## Conclusion

In this study, we used weak supervision and three traditional machine learning model: logistic regression, random forest, and SVM, and six different BERT models: Bert base, PubmedBERT (Abs + Ft), Pubmed-BERT (Abs), Bio BERT, UMLS BERT, and Bio-clinical BERT, to classify the lifestyle status in AD from clinical notes. The purpose of using weak supervision was to prevent the need for the laborious task of creating a hand-labeled dataset. In addition, we evaluated two text classification case studies’ effectiveness: classifying sentences regarding their physical activity and excessive diet. UMLS BERT and Bioclinical BERT model performed the best for the two use cases. The study group can further expand this approach to other factors such as substance abuse to investigate their effects on AD and provide additional AD research opportunities.

## Data Availability

Due to the privacy issues, the data used in this study are not publicly available.

## Abbreviations

AD: Alzheimer’s disease
BERT: Bidirectional Encoder Representations from Transformer
CUIs: Concept Unique Identifiers
GSC: Gold Standard Corpus
MIMICIII: Medical Information Mart for Intensive Care III
SVM: Support Vector Machine
UMLS: Unified Medical Language System
